# Enhancing Humoral Responses Against HIV Envelope Trimers via Nanoparticle Delivery with Stabilized Synthetic Liposomes

**DOI:** 10.1038/s41598-018-34853-2

**Published:** 2018-11-08

**Authors:** Talar Tokatlian, Daniel W. Kulp, Andrew A. Mutafyan, Christopher A. Jones, Sergey Menis, Erik Georgeson, Mike Kubitz, Michael H. Zhang, Mariane B. Melo, Murillo Silva, Dong Soo Yun, William R. Schief, Darrell J. Irvine

**Affiliations:** 1 Koch Institute, MIT, Massachusetts, USA; 2 International AIDS Vaccine Initiative, New York, USA; 3 Center for HIV/AIDS Vaccine Immunology and Immunogen Discovery, The Scripps Research Institute, California, USA; 4 Vaccine and Immunotherapy Center, The Wistar Institute, Pennsylvania, USA; 5 Immunology and Microbial Science, TSRI, California, USA; 6 Department of Biological Engineering and Materials Science & Engineering, MIT, Massachusetts, USA; 7 Ragon Institute of MGH, MIT, and Harvard, Massachusetts, USA; 8 Howard Hughes Medical Institute, Maryland, USA

## Abstract

An HIV vaccine capable of eliciting durable neutralizing antibody responses continues to be an important unmet need. Multivalent nanoparticles displaying a high density of envelope trimers may be promising immunogen forms to elicit strong and durable humoral responses to HIV, but critical particle design criteria remain to be fully defined. To this end, we developed strategies to covalently anchor a stabilized gp140 trimer, BG505 MD39, on the surfaces of synthetic liposomes to study the effects of trimer density and vesicle stability on vaccine-elicited humoral responses in mice. CryoEM imaging revealed homogeneously distributed and oriented MD39 on the surface of liposomes irrespective of particle size, lipid composition, and conjugation strategy. Immunization with covalent MD39-coupled liposomes led to increased germinal center and antigen-specific T follicular helper cell responses and significantly higher avidity serum MD39-specific IgG responses compared to immunization with soluble MD39 trimers. A priming immunization with liposomal-MD39 was important for elicitation of high avidity antibody responses, regardless of whether booster immunizations were administered with either soluble or particulate trimers. The stability of trimer anchoring to liposomes was critical for these effects, as germinal center and output antibody responses were further increased by liposome compositions incorporating sphingomyelin that exhibited high *in vitro* stability in the presence of serum. Together these data highlight key liposome design features for optimizing humoral immunity to lipid nanoparticle immunogens.

## Introduction

Despite recent global declines in HIV infection and AIDS-related deaths as a result of the increasing availability of anti-retroviral drugs, HIV remains a widespread pandemic with over 36 million living with HIV/AIDS as of 2016^1^. To finally eliminate new HIV infections a prophylactic vaccine remains an urgent need. It is likely that a successful vaccine against HIV-1 will need to induce broadly neutralizing antibodies (bnAbs) that neutralize diverse strains of the virus^2–4^. Isolated bnAbs from rare individuals who are able to naturally control infection have been shown to target conserved regions of the HIV envelope spike on HIV, which is comprised of 3 copies of the subunits gp120 and gp41 non-covalently assembled into trimers. Passive transfer of bnAbs into non-human primates protects from challenge with simian immunodeficiency virus expressing HIV-1 envelopes^5–8^. In order to elicit such antibodies by immunization, recombinant env immunogens recapitulating the structure of the native trimer have been avidly pursued. These efforts have led to the generation of recombinant soluble trimers that preserve native neutralizing conformational epitopes targeted by many known bnAbs but which sequester epitopes commonly recognized by non-neutralizing antibodies^9–14^. A particularly successful strategy for generation of native-like gp140 trimers has been through the design of so-called SOSIP immunogens, fully cleaved trimers stabilized by gp120-gp41 disulfide bonds and mutation of critical residues promoting interactions between gp41 subunits^9,15,16^. Additional design modifications have further improved the antigenic profiles of native-like trimers^13,17,18^.

Recent successes in employing SOSIP trimers to elicit for the first time autologous tier 2 neutralizing antibody titers in rabbits^19^ and non-human primates^20^ have spurred enthusiasm for exploring new avenues to further promote humoral responses to these immunogens. One such avenue is the use of nanoparticles for the multivalent display of trimers, which could promote B cell activation and engagement of low affinity B cell precursors through enhanced B cell receptor (BCR) crosslinking. It has been widely demonstrated that particulate display of antigens, in the forms of virus-like particles, synthetic nanoparticle platforms, or protein/peptide nanoparticles and nanocarriers, can significantly boost humoral responses and the development of protective antibody responses compared to monomeric immunogens^21–26^. In the context of HIV vaccine development, both protein- and lipid-based nanoparticles have previously been reported and shown to enhance humoral responses to trimer immunogens^27–29^. Alternatively, unilamellar liposomes have a well-established track record as safe and effective drug delivery vehicles in humans, as demonstrated by currently approved liposome-based drugs (e.g. Doxil, Myocet, and Marqibo for cancer therapy, DepoDur for pain management, Ambisome for treatment of fungal infections, and several others) and formerly approved vaccines (e.g. Epaxal® for hepatitis A and Inflexal® V for influenza; both were discontinued in 2014 due to manufacturing quality issues)^30^. Given their ability to be prepared in a range of particle sizes and capacity to be conjugated to any antigen of interest, we and others have demonstrated that stabilized trimers can be densely conjugated to the surface of unilamellar liposomes using both non-covalent^18,31^ and covalent^20,32^ coupling strategies.

Here we explored properties of a liposomal nanoparticle platform that allows for both oriented and stable conjugation of HIV envelope trimers. Trimer-conjugated liposomes improved both germinal center (GC) B cell and trimer-specific T follicular helper (T_fh_) cell responses, resulting in significantly higher trimer-specific antibody titers and overall higher avidity antibody production relative to immunization with soluble MD39 SOSIP trimers. Additional optimization of the liposome composition to improve stability in the presence of serum was shown to even further enhance germinal center responses in mice. These results define key design criteria for the generation of effective lipid nanoparticle immunogens for HIV and other infectious diseases.

## Results

### Synthesis of liposome-based HIV trimer virus-like particles

**Figure 1:**
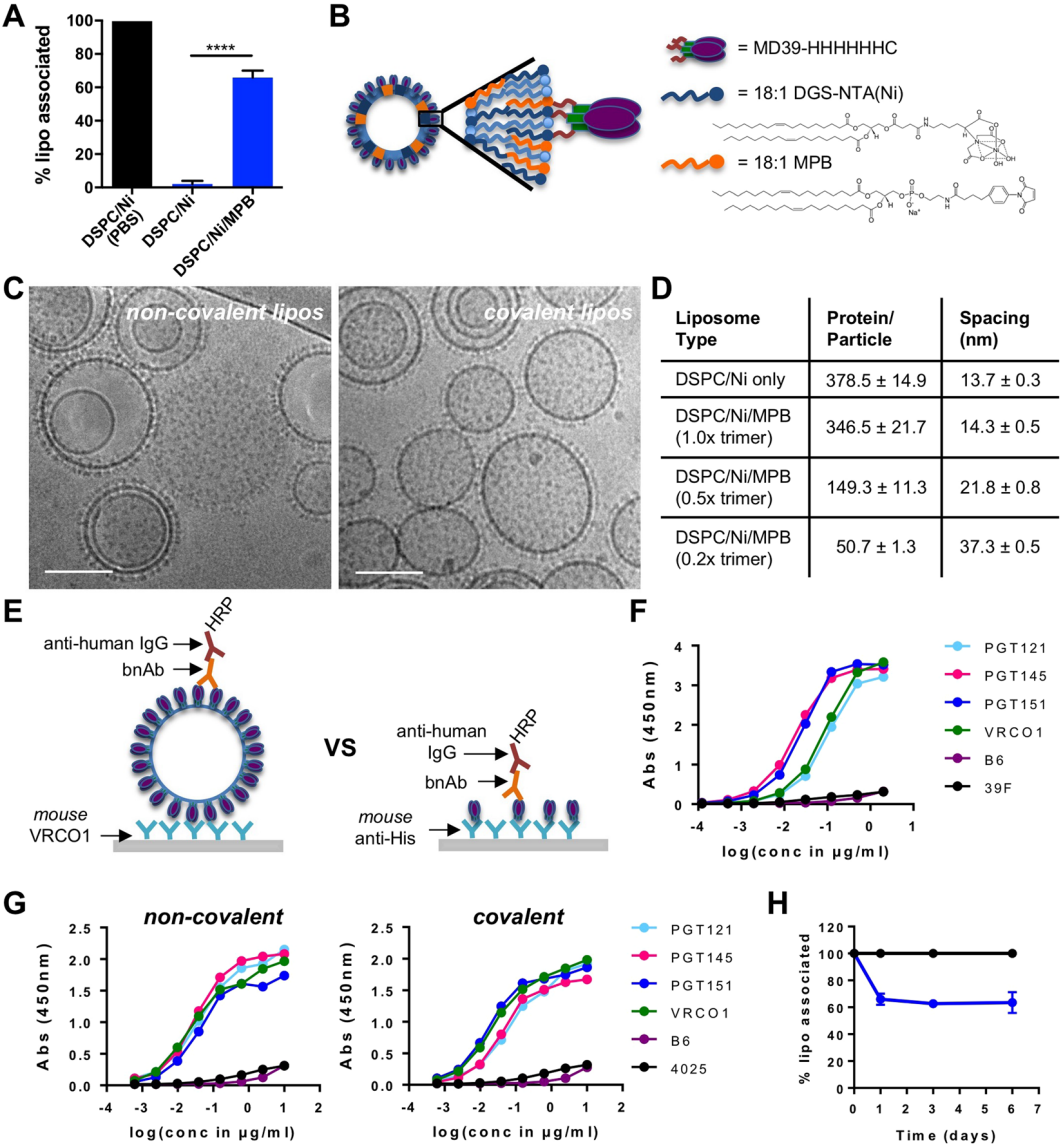
Synthesis of covalently-anchored trimer-liposomes. (**A**) Serum stability analysis comparing the fraction of MD39 trimer retained on liposomes prepared by covalent coupling or Ni-NTA binding following 24 hours incubation in 10% mouse serum (blue bars) at 37 °C (n = 3–4; ****p < 0.0001, *t* test). (**B**) Covalent coupling strategy. (**C**) Representative cryoEM images of non-covalent and covalently coupled MD39 liposomes. Scale bar = 100 nm. (**D**) Trimer densities on 150 nm-diameter liposomes and center-to-center spacing as a function of coupling strategy and relative trimer concentration during liposome conjugation. (**E**) Schematic of antigenic profile analysis on intact liposomes compared to soluble trimers. bnAb (PGT121, PGT145, PGT151, VRC01) and non-nAb (B6, 39 F, 4025) binding profiles of (**F**) soluble MD39 trimers and (**G**) non-covalent and covalent MD39 liposomes. (**H**) Retention of MD39 on covalent liposomes over time in PBS (black) or 10% mouse serum (blue) at 37 °C (n = 3).

To generate highly multivalent nanoparticles that could in theory be employed for delivery of any trimer immunogen while meeting criteria for clinical translation (scalable manufacturability, safety/biodegradability^30^), we focused on unilamellar liposomes. As model immunogens, we employed a SOSIP trimer known as BG505^16^, or a stabilized version of this trimer called MD39, which incorporates multiple mutations designed to limit sampling of non-native conformations, such as those that expose the V3 loop, and to increase expression and thermal stability^13,18^. We previously demonstrated that mixing of MD39 trimers containing C-terminal histags on each gp140 subunit with liposomes functionalized with Ni-NTA headgroup lipids leads to oriented anchoring of the immunogen to the vesicle surfaces^18^. To produce high density trimer-liposomes with a high overall coupling efficiency (~95%), we found the optimal ratio of MD39-6xHis:Ni-NTA during coupling to be approximately 1:40. However, despite the availability of 3 histags per trimer for Ni-NTA anchoring, when incubated with 10% mouse serum for 24 hours, these non-covalently anchored trimers were completely dissociated (Fig. 1A and Supplementary Fig. S1), similar to what has been previously described by others^33–35^. To address this issue, liposomes were prepared with 5 mol% maleimide-functionalized lipids (MPB) in addition to 5 mol% Ni-NTA lipids, and MD39 trimers were modified to introduce a single free cysteine residue immediately adjacent to each histag at the C-terminus of the gp41-ectodomain trimer subunits (Fig. 1B). Efficient trimer coupling was obtained by including both Ni-NTA (to rapidly capture the trimers on the vesicle surfaces) and MPB (to subsequently covalently react with the C-terminal trimer cysteines). CryoEM imaging revealed similar morphologies for liposomes loaded with covalently- or non-covalently-anchored trimers, and revealed oriented attachment of trimers across the liposome surfaces (Fig. 1C and Supplementary Fig. S2A,B). Further titrating the MD39:lipid ratio during the initial conjugation step allowed for the density of trimer coupled to the liposome surfaces to be readily controlled (Fig. 1D and Supplementary Fig. S2C). Except where otherwise noted, we focused our analyses on liposomes decorated with the maximum density of trimer (~14 nm center-to-center spacing between trimers on the liposome surfaces) with a mean vesicle diameter of ~150 nm (Supplementary Fig. S2D). Importantly, MD39 trimer antigenicity, determined by ELISA analysis of monoclonal antibody binding to liposomes captured on plates coated with the CD4 binding site-directed antibody VRC01 (Fig. 1E), was similar between the initial MD39 protein and liposome-conjugated trimer regardless of coupling strategy. MD39-liposomes showed high levels of binding by bnAbs (PGT121 (V3 glycan specific bnAb), PGT145 (V1/V2 glycan specific bnAb), PGT151 (gp120/gp41 interface specific bnAb), VRC01 (CD4 binding site specific bnAb)) and minimal binding by non-nAbs (B6 (CD4 binding site specific non-nAb), 4025 or 39 F (both V3 specific non-nAbs); Fig. 1F,G). As expected, introduction of covalent linkages via MBP lipids enhanced the retention of trimer on liposomes exposed to serum, with ~60% of the initially loaded trimer retained following incubation with 10% serum for 24 hr (Fig. 1A). Trimers retained after the rapid initial loss (<1 day) then remained stably attached for at least one week in serum (Fig. 1H). Thus, covalent anchoring allowed trimers to be loaded at high density on liposomes, retaining a native-like antigenicity profile, while improving serum stability of the particulate immunogen.

**Figure 2:**
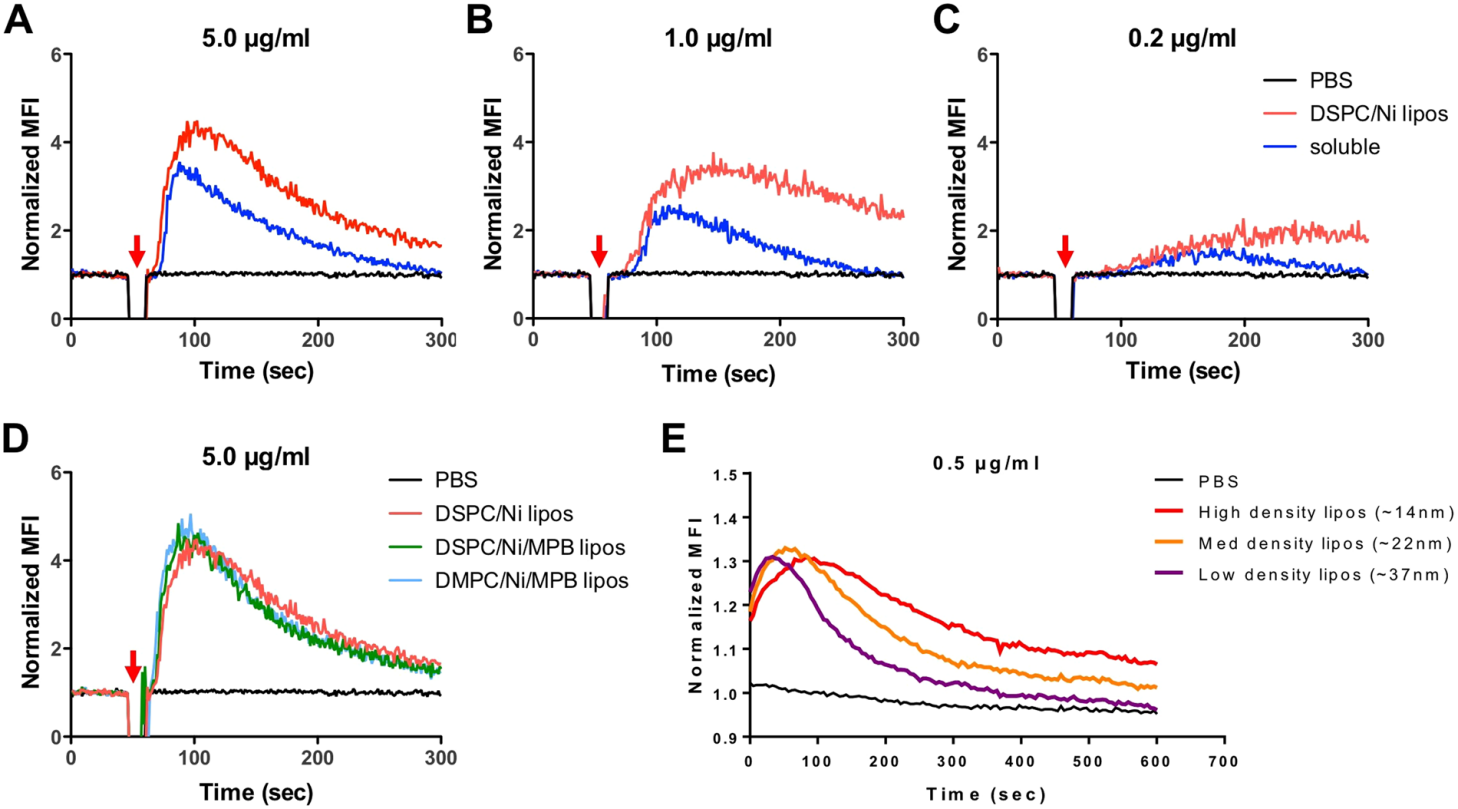
Multivalent display of MD39 trimers from liposomes enhances activation of VRC01-expressing B cells *in vitro*. (**A–C**) Comparison of calcium flux by VRC01-expressing B cells in response to soluble or multivalent display of trimers at (**A**) 5, (**B**) 1 or (**C**) 0.2 µg/ml of trimer. (**D**) Comparison of calcium flux in response to multivalent display of trimers on various liposomes at 5 µg/ml of trimer. (**E**) Extended *in vitro* activation of VRC01-expressing B cells with liposomes with various densities of MD39 trimer at 0.5 µg/ml trimer. Center-to-center spacing of trimers on various liposomes denoted within parentheses. Data shown is representative of 2–3 separate experiments.

We previously demonstrated that liposomes conjugated with a high density of engineered germline-targeting trimers were able to activate B cells expressing a germline B cell receptor with very low affinity (K_D_ > 10 µM) for gp140 at 1000-fold lower concentrations compared to soluble trimers^18^, suggesting a substantial advantage for nanoparticulate immunogens in a primary immune response. However, repeated booster immunizations as commonly employed in HIV vaccine studies will promote the development of B cells with high affinity for the antigen, and it is unclear if particulate display will still be important in this setting. To shed light on this issue, we assessed the activation of B cells expressing the high-affinity CD4 binding site antibody VRC01 in a calcium flux assay. Despite the higher affinity of VRC01 for the MD39 trimer (K_D_ ~ 124 nM)^13^, MD39-liposomes triggered stronger calcium signaling than soluble trimer across a range of concentrations, eliciting a greater peak signaling intensity at all trimer concentrations tested (Fig. 2A–C). Addition of MD39-liposomes to VRC01 B cells led to similarly strong activation of calcium signaling whether trimers were conjugated by Ni-NTA/histag binding alone or covalent coupling (Fig. 2D). We tested the importance of membrane rigidity by preparing liposomes with DSPC (T_m_ = 55 °C) or DMPC (T_m_ = 24 °C) phospholipids, but these particles exhibited identical B cell stimulation (Fig. 2D). Activation of B cells by nanoparticle-displayed trimers was influenced by trimer density, with more sustained signaling triggered by liposomes bearing high-density trimer (Fig. 2E). Thus, high-density liposome display enhances B cell activation at the single cell level.

### Immunogenicity of covalent MD39-coupled liposomes in mice

**Figure 3:**
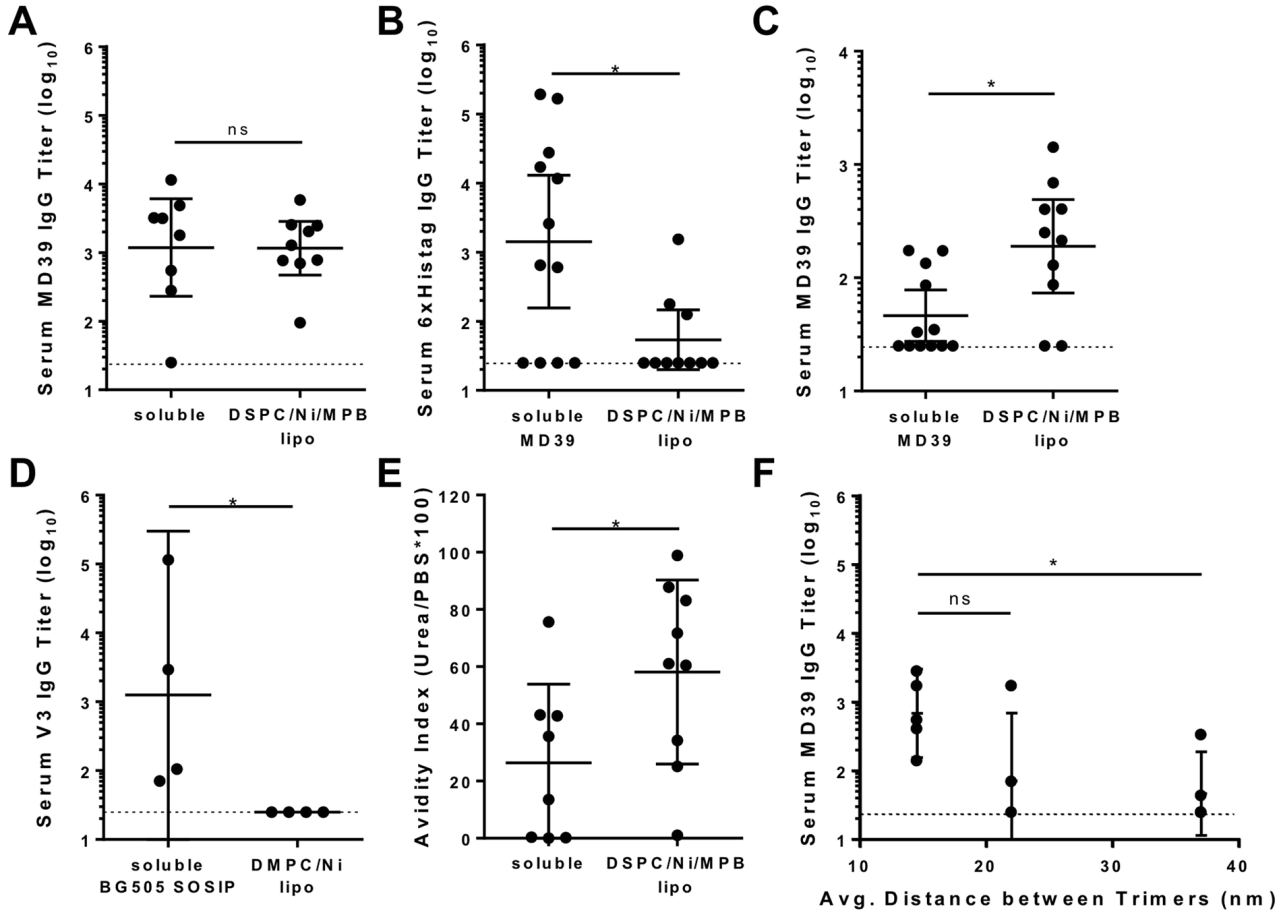
MD39-liposomes elicit higher avidity trimer-focused antibody responses compared to soluble MD39 trimer immunization. Humoral responses in mice 3 weeks post-boost. Balb/c mice were immunized with 1 µg MD39 trimer and 0.2U ISCOMATRIX, and boosted at 6 weeks with the same formulations. Data shown contains pooled samples from 2 or 3 replicate experiments with each individual experiment having 3–5 animals/group. (**A**) Serum MD39–6xhis-specific titers (*n* = 8–12 animals/group). (**B**) Serum 6xhis tag-specific titers. (**C**) Serum MD39-specific titers determined by ELISA using non-6xhis tagged MD39 trimers to exclude histag-specific responses. (**D**) Serum V3-specific titers following immunization with 2 µg wild-type BG505 SOSIP trimers and 1 µg MPLA co-delivered either in soluble form or within non-covalent SOSIP liposomes (*n* = 4 animals/group). (**E**) Avidity of polyclonal antibodies from immunization with MD39 and ISCOMATRIX (from part **A**). (**F**) Serum MD39-6xhis-specific titers following immunization with MD39 on covalent liposomes at varying surface densities with ISCOMATRIX adjuvant (*n* = 5 animals/group). X-axis denotes average center-to-center trimer spacing on 150 nm liposomes. Dotted line in titer plots denotes limit of detection. *p < 0.05, Mann-Whitney test for titers and unpaired *t* test for avidity. Panel F was analyzed by a Kruskal-Wallis test.

We next compared in detail the humoral responses elicited by soluble trimers vs. covalently-anchored liposomal trimers. Balb/c mice were primed on day 0 and boosted at 6 weeks with 1 µg of MD39 trimer (soluble or on liposomes) and ISCOMATRIX^36^, and serum IgG responses were analyzed 3 weeks post-boost. ISCOMATRIX adjuvant, which was first described by Morein *et al*.^37^, is a 40 nm cage-like nanoparticle composed of lipids, cholesterol, and Quil A saponin and posses both innate and adaptive TLR-independent immune modulation and antigen delivery characteristics^38^. Immunization with MD39 trimer-liposomes or soluble MD39 trimer resulted in comparable MD39-6xHis-specific IgG titers (Fig. 3A). However, soluble MD39 induced a high level of irrelevant anti-histag antibodies, whereas histag-directed responses were undetectable in 7/10 liposome-immunized animals (Fig. 3B). We speculate this altered specificity is due to steric sequestration of the trimer histags against the liposome surface. Consistent with a histag-dominated response to the soluble trimer, ELISA analysis of serum IgG binding to MD39 trimers lacking histags revealed a 4.1-fold mean difference in trimer-specific response by immunization with covalent liposomes compared to soluble trimer, with many of the soluble trimer-immunized animals showing no *bona fide* trimer-specific response (Fig. 3C, p = 0.0138). These responses were sustained over several months, despite only the single booster immunization after 6 weeks (Supplementary Fig. S3). Immunization with non-covalent liposomes bearing Ni-NTA-anchored trimers similarly produced strong antibody titers (Supplementary Fig. S4A). However, as expected due to their instability in the presence of serum proteins, these liposomes also elicited high levels of histag-specific antibodies comparable to immunization with soluble trimers (Supplementary Fig. S4B). In addition to the synthetic protein tag, the V3 loop is a highly immunodominant epitope that is not exposed in trimers with a native-like, closed conformation^17,19^. V3-loop specific responses were not detected in the majority of mice immunized with MD39 (soluble or on covalent liposomes) (Supplementary Fig. S5), likely due to the engineered stability of the MD39 trimer^13^. In contrast, in a separate immunization study with wild-type BG505 SOSIP, V3-loop responses were elicited by soluble BG505 immunization, and these responses were completely eliminated when BG505 trimer was anchored to liposomes (Fig. 3D).

Measurement of the avidity of antibodies elicited by trimer immunization using a mild urea treatment to disrupt low affinity antigen-antibody interactions during ELISA analysis revealed that soluble trimer immunization elicited low-avidity antibody responses in a majority of animals, but liposome delivery elicited significantly higher avidity responses (Fig. 3E). One key observation was that this high avidity response was only observed with more rigid liposomes comprised mainly of DSPC lipids, while liposomes that were expected to be more fluid and containing mainly DMPC were not able to elicit high avidity responses *in vivo* (Supplementary Fig. S6) despite equivalent activation of antigen-specific B cells *in vitro* (Fig. 2D). Trimer density on the liposome surfaces also impacted the magnitude of the humoral response, with the highest IgG responses elicited by maximally-packed trimers on the liposome surfaces (Fig. 3F).

### Liposomal delivery enhances germinal center responses to trimer immunization

**Figure 4:**
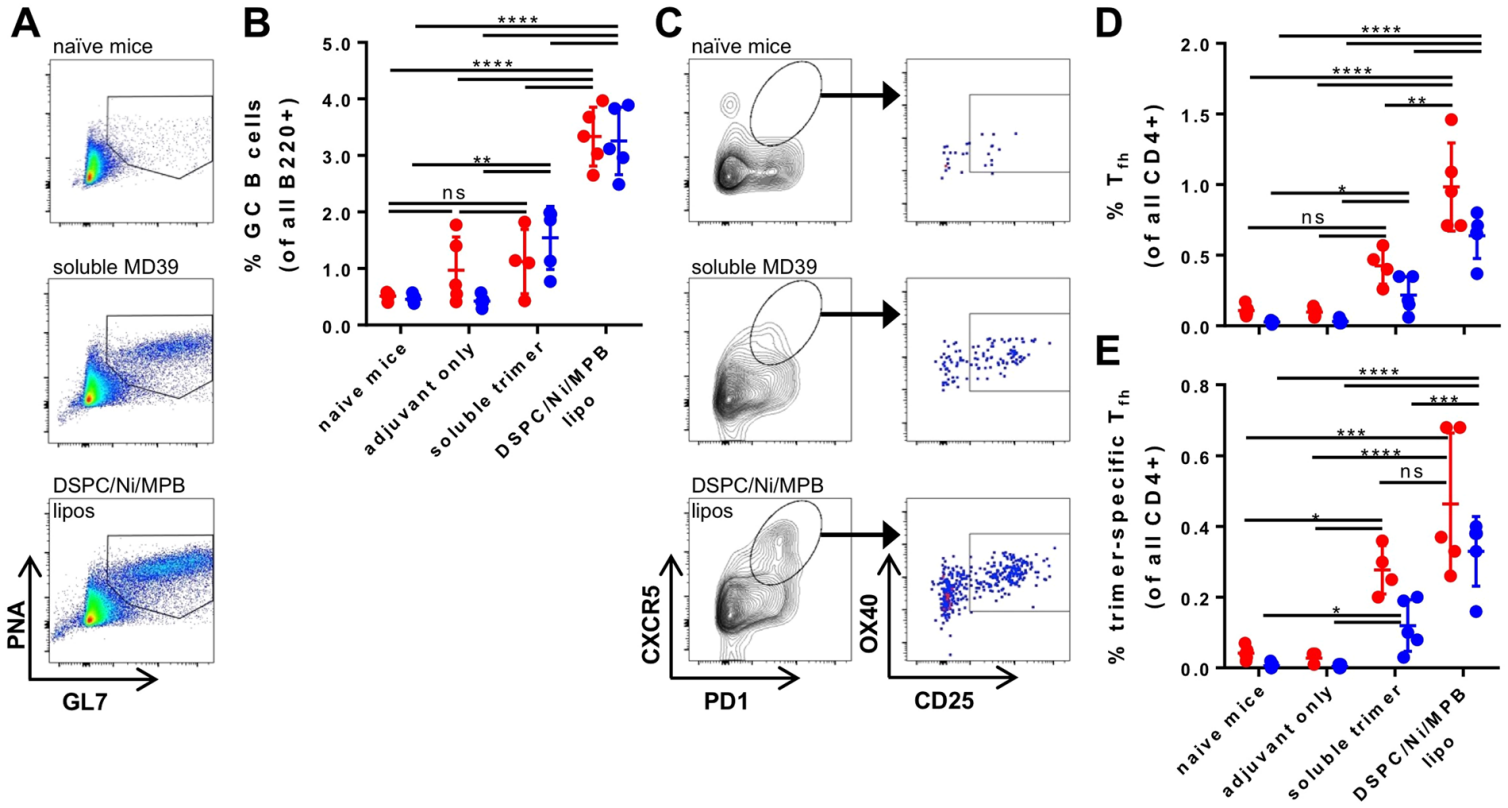
Trimer-liposomes elicit stronger germinal center responses than soluble trimer immunization. (**A**) Representative flow plots of germinal center B cell populations (parent gate B220+ IgD-low). (**B**) GL7+ PNA+ IgD-low (B220+) GC B cell frequency in draining inguinal lymph nodes at 7 (red) and 21 (blue) days post-immunization. (**C**) Representative flow plots of total (CXCR5+ PD1+ with parent gate CD4+ CD44+) T_fh_ and antigen-specific (OX40+ CD25+ PDL1+) T_fh_ after 18 hour *ex-vivo* peptide stimulation. Frequencies of (**D**) total and (**E**) antigen-specific T_fh_ in draining inguinal lymph nodes at 7 (red) and 21 (blue) days post-immunization. Each dot in plots (**B**,**D**,**E**) represents one animal (n = 4–5 animals/group); *p < 0.05, **p < 0.01, ***p < 0.001, ****p < 0.0001, ordinary one-way ANOVA with Tukey post-test on each time point.

The high degrees of somatic hypermutation and unusual structural features characteristic of broadly neutralizing antibodies will likely require highly active and long-lived germinal centers (GCs) to be stimulated by a successful HIV vaccine. To assess the impact of liposome delivery on GC responses *in vivo*, GC B cells in draining inguinal lymph nodes were enumerated by flow cytometry at 7 and 21 days following immunization (Fig. 4A). MD39-liposome immunization approximately tripled the frequency of GL7^+^PNA^+^ germinal center B cells compared to immunization with soluble trimer 7 days after immunization, and sustained this difference at 3 weeks post-prime (Fig. 4B). We next enumerated MD39-specific follicular helper T cell (T_fh_) responses at the same time points, using a recently described activation-induced marker assay (Fig. 4C)^39^. While differences in total T_fh_ were significant at both 7 and 21 days, responses trended toward at least 2-fold more trimer-specific T_fh_ at any given time after immunization with covalent trimer-conjugated liposomes compared to soluble MD39 (Fig. 4D,E). Adjuvant alone (Fig. 4B,D,E) or blank liposomes with or without Ni-NTA lipids (Supplementary Fig. S7) elicited no detectable GC B cell or Tfh responses above background levels observed in naïve, unimmunized mice. Thus, liposomal vaccination enhanced both T_fh_ and GC B cell induction compared to soluble trimer in primary immunizations.

### Particulate priming is especially important for inducing high avidity antibodies

**Figure 5:**
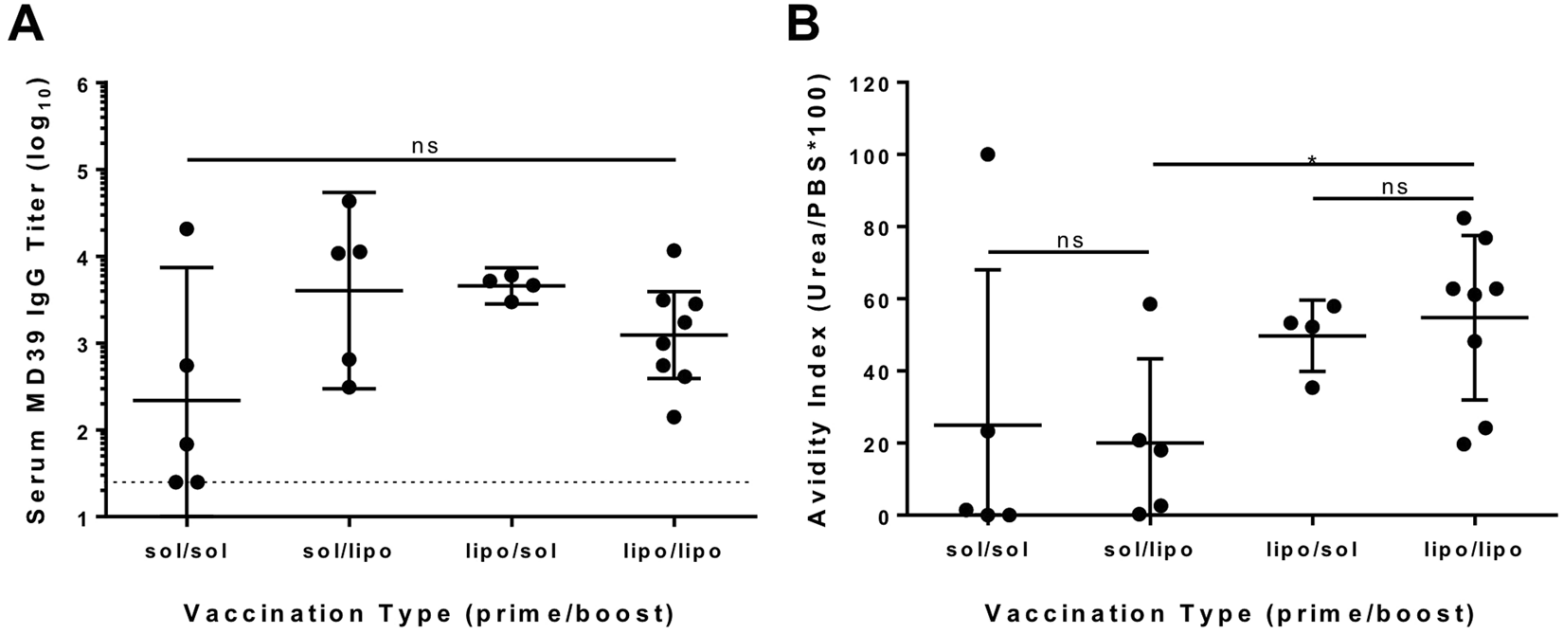
Liposomal trimer delivery maximizes humoral responses during primary immunization but less critical druing booster immunizations. Balb/c mice were immunized with 1 µg MD39 trimer and 0.2U ISCOMATRIX, and boosted at 6 weeks with the same formulations. (**A**) Serum MD39-6xhis-specific IgG titers (*n* = 3–4 animals/group); dotted line denotes limit of detection. (**B**) Avidity of polyclonal antibodies. Avidity was analyzed using independent unpaired *t* tests for direct comparisons; *p < 0.05.

In a primary immune response, particulate delivery of immunogens should provide a clear advantage over immunization with soluble/monomeric antigen. By contrast, in secondary responses stimulated by booster immunizations, the presence of pre-existing IgM/IgG will lead to the rapid formation of immune complexes of antibodies with injected immunogens, potentially converting even soluble antigen into particulates *in situ*, possibly limiting the benefits of nanoparticle delivery for boosting. To assess whether liposomal delivery was more important during priming vs. boosting, groups of balb/c mice were immunized with trimer in liposomal form during the prime only, the boost only, or during prime and boost, compared to a traditional immunization with trimer in soluble form only. As shown in Fig. 5A, MD39-6xHis-specific IgG titers were comparable between all immunization groups following booster immunization. However, when the avidities of these IgG responses were compared, immunization that included liposomes during priming appeared to exhibit increased binding strength compared to the vaccinations using soluble trimer as the prime (Fig. 5B). Thus, compared to soluble trimers, particulate delivery appears to initiate a more effective primary immune response that is sustained post boosting.

### Further enhancing liposome stability improves humoral responses

**Figure 6:**
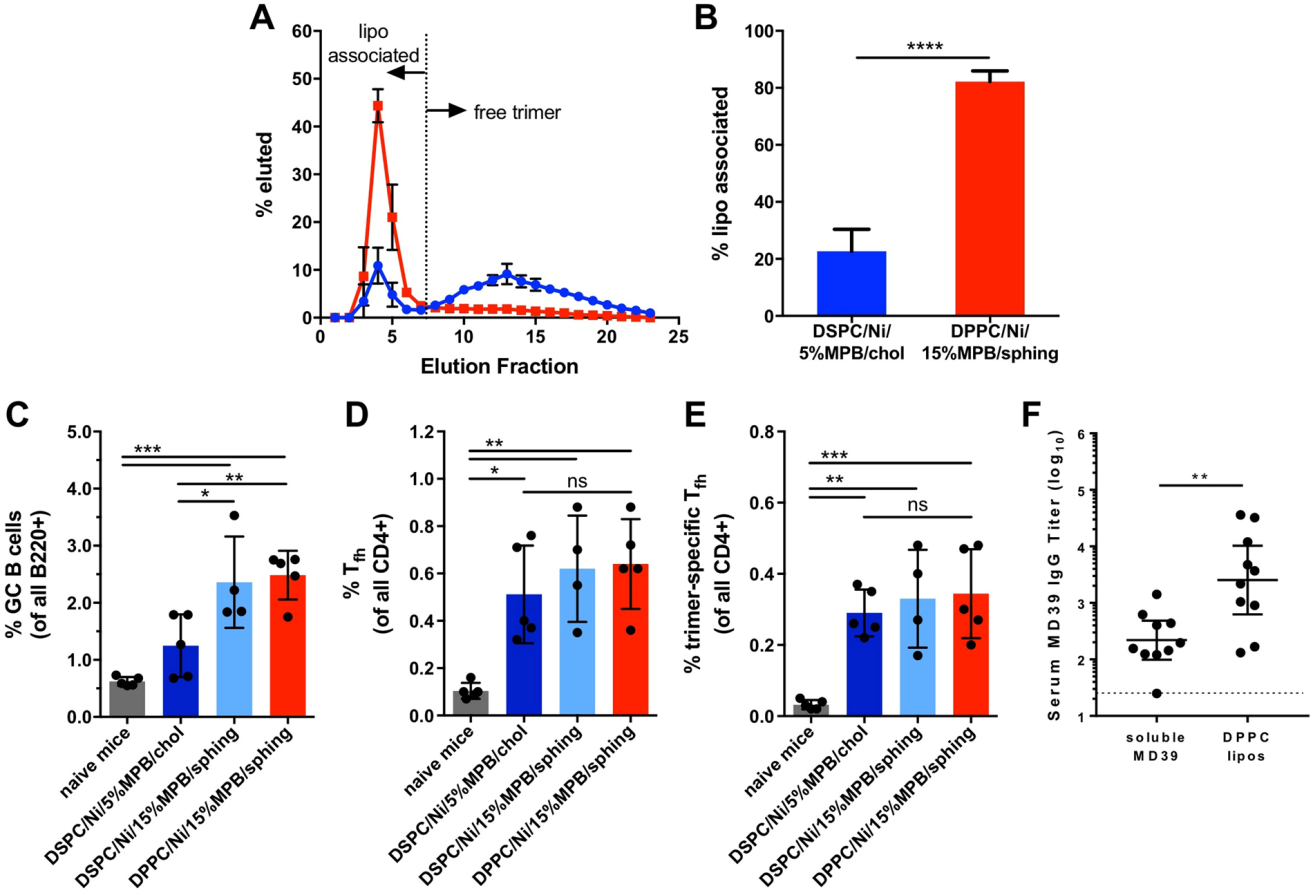
Optimizing *in vitro* liposomal trimer serum stability leads to enhanced germinal center and IgG responses *in vivo*. (**A**,**B**) Serum stability analysis comparing covalent MD39 trimer conjugated liposomes formed with DSPC:Ni-NTA:MPB:cholesterol (61.5:5:5:28.5; blue) or DPPC:Ni-NTA:MPB:sphingomyelin (51.5:5:15:28.5; red) after 3 days in 20% mouse serum at 37 °C (n = 3–4; ****p < 0.0001). (**A**) Size exclusion chromatography profile of liposomes and (**B**) quantification of remaining liposome-associated trimer after serum treatment. (**C–E**) Germinal center responses following immunization with increasingly stabilized trimer-conjugated liposomes. Frequencies of (**C**) GL7+ PNA+ IgD-low (B220+) GC B cells, (**D**) total (CXCR5+ PD1+ with parent gate CD4+ CD44+) and (**E**) antigen-specific (OX40+ CD25+ PDL1+) T_fh_ in draining inguinal lymph nodes 7 days post-immunization (*n* = 5 animals/group). *p < 0.05, **p < 0.01, ***p < 0.001, ordinary one-way ANOVA with Tukey post-test. (**F**) Serum MD39-specific titers in mice 3 weeks post-boost comparing immunization with MD39 trimers either in soluble form or highly stabilized liposomes. Mice were immunized with 1 µg MD39 trimer and 5 µg in-house formulated saponin adjuvant, and boosted at 6 weeks (*n* = 10 animals/group). MD39-specific titers were determined by ELISA using non-6xhis tagged MD39 trimers to exclude histag-specific responses; dotted line denotes limit of detection. **p < 0.01, Mann-Whitney test.

The data discussed above demonstrated enhanced GC responses, T_fh_ priming, and output antibody titers to trimers delivered in liposomal vs. soluble form, despite the liposome formulation only exhibiting modest stability in serum *in vitro* (Fig. 1A). Thus, we finally explored whether optimizing the liposomal membrane composition for greater serum stability could promote further enhancements in trimer immunogenicity. To this end we tested increasing the amount of maleimide-headgroup lipids included in the liposomal bilayers and introducing the hydrogen-bonding lipid sphingomyelin and/or the lower-T_m_ lipid DPPC (T_m_ = 41 °C) into the liposome bilayers to improve trimer coupling and increase bilayer stability, respectively. It was quantitatively and qualitatively confirmed by ELISA and cryoEM, respectively, that overall trimer density was unaffected despite changes in liposome composition. To assess the impact of these altered liposome compositions on serum stability, we incubated MD39-liposomes in 20% mouse serum for 3 days and then separated intact liposomes from free trimer by size exclusion chromatography. This stringent test was used to more closely mimic protein levels in lymphatic fluid^40,41^. Increasing maleimide-lipid content from 5 to 15%, exchanging cholesterol for sphingomyelin, and exchanging DSPC for DPPC all incrementally improved the stability of trimer-liposomes in serum, with ~80% trimer retention after 3 days in 20% mouse serum for the most stable composition (Fig. 6A,B and Supplementary Fig. S8).

Increasing stability of the liposomes correlated with improved early germinal center responses *in vivo*, with covalent trimer-conjugated liposomes containing DPPC and sphingomyelin resulting in significantly higher GC B cell frequencies compared to the original liposome formulation (Fig. 6C). Here soluble trimers or trimer-conjugated liposomes were co-injected with an in-house generated formulation of saponin nanoparticles in place of ISCOMATRIX (due to the lack of access to commercial ISCOMATRIX at the time of this study). Interestingly, frequencies of both total and trimer-specific T_fh_ remained unchanged as a function of overall liposome stability (Fig. 6D,E). Given these trends in GC induction, we next compared immunization of the most stable DPPC liposome formulation directly with soluble trimers. Stable trimer-conjugated DPPC liposomes elicited significantly higher titers (11.6-fold greater) compared with soluble trimers 3 weeks post-boost (Fig. 6F, p = 0.0052), a greater relative increase than that observed with the original liposome formulation (Fig. 3C), albeit with a slightly modified adjuvant.

## Discussion

While the benefits of multivalent antigen delivery for vaccines have been widely documented, studies to evaluate nanoparticle delivery of HIV env trimers have only gained momentum within the last few years. Across several different platforms, particulate trimer immunogens with increased valency have been shown to trigger both germline and mature B cell signaling more strongly compared to soluble gp140 molecules^18,32,42^, despite the inherently trimeric nature of the HIV env complex. Liposomes are an attractive platform for nanoparticle delivery of HIV immunogens, as many liposomal products have successfully been translated into the clinic and the tunable size of lipid vesicles enables as many as several hundred (300+) trimers to be displayed on a single particle, with controllable density. Liposomes decorated with trimers spaced ~14 nm apart were able to strongly activate VRC01-expressing B cells *in vitro* irrespective of liposome composition and trimer conjugation strategy (Fig. 2), in agreement with previous reports^32^. Reduced valency and increased trimer spacing negatively impacted B cell activation *in vitro*, but these trends were not as clearly evident *in vivo* likely due to the many additional steps beyond BCR engagement involved in the immune response in lymphoid tissues. However, immunization with liposomes bearing trimers at a nearly close-packed spacing of 14 nm tended to increase antibody titers compared to immunization with soluble antigen. These results are directly in line with the understanding that HIV has evolved to only express a low number of intact envelope trimer copies (~14/HIV) to evade bivalent binding by IgG and limit the rate of broadly neutralizing antibody induction^43^.

While not fully validated in the context of neutralizing antibody responses for HIV, the current consensus is that removal of potentially immunologically distracting, non-neutralizing epitopes may improve responses to other more desirable epitopes^17,19^. Covalent conjugation nearly eliminated recognition of proximal histags and potentially helped to maintain trimer stability *in vivo*, as demonstrated by reduced V3-eptiope specific responses for a native BG505 trimer with relatively high V3 exposure compared to MD39 (Fig. 3D). Covalent display of trimers also resulted in significantly higher germinal center and sustained antigen-specific T_fh_ responses. Strong T_fh_ responses are essential in driving high affinity antibody responses in immunization, as exemplified by the significantly higher avidity antibody responses that were produced upon immunization with covalent liposome coupled trimers. Notably, we found that the nanoparticle form of trimers was most important during the initial prime immunization, with lower impact during boosting. Yet despite differences in antibody avidity, overall antibody titers were not significantly different as immunization with soluble trimers especially resulted in highly variable titer responses.

Pauthner and Havenar-Daughton and colleagues recently showed induction of neutralizing antibodies in non-human primates (NHPs) which was attributed to the use of stabilized trimers in combination with an improved immunization schedule and route of administration^20^. Unexpectedly, however, multivalent display of trimers in this study minimally increased MD39 SOSIP-specific titers and did not improve induction of neutralizing responses. As revealed by the present studies, this finding was likely a result of poor overall particle stability in the presence of serum proteins. Additional studies by Wyatt and colleagues showed covalent conjugation of JRFL trimers to liposomes could induce high avidity neutralizing antibodies in some NHPs^44^. As such, a better understanding of how stable, multivalent delivery of HIV env trimers triggers early germinal center responses and initiates induction of high affinity antibodies will be critical for future vaccine design.

Covalent conjugation of MD39 trimers to liposomes composed of 61.5% DSPC, 28.5% cholesterol, 5% Ni-NTA, and 5% MPB improved serum stability significantly compared to Ni only liposomes (~60% retention compared to ~5% after 1 day in 10% serum). While these liposomes clearly demonstrated improvements over immunization with soluble trimers in mice, they may still not have been stable enough to fully capture the potential of high-density display of HIV env trimers to engage antigen-specific B cells *in vivo* and induce strong neutralizing responses in NHPs^20^. We finally tested several liposome formulations (Supplementary Fig. S8) and each lipid component was determined to be critical in controlling either overall liposome stability or conjugation between the trimer and liposome. Increasing the maleimide content from 5 to 15% improved trimer retention in serum, although increasing past 15% did not provide further enhancements (Supplementary Fig. S8A). Importantly, 5% Ni-NTA lipids were still necessary to maintain overall coupling efficiency (>95%). Other strategies to improve coupling, such as introducing an optimal length linker between the trimer and the reactive end group as described by Bale *et al*.^32^, could also be useful, although they were not tested in this study. Cholesterol has been widely demonstrated to tighten phospholipid bilayers, especially in combination with gel-phase phospholipids^45,46^. However, exchanging cholesterol for sphingomyelin significantly improves bilayer stability by introducing inter- and intra-molecular hydrogen bonding^46^. Finally, exchanging DSPC for DPPC further enhanced the stability of sphingomyelin-containing liposomes, perhaps due to improved compatibility of DPPC and sphingomyelin. Importantly, these substitutions resulted in highly stable trimer-liposome particles (final composition: 51.5% DPPC, 28.5% sphingomyelin, 5% Ni-NTA, and 15% MPB) that exhibited improved early GC responses and significantly higher overall trimer-specific titers.

As described above, several findings in this study are in agreement with those reported by Bale and colleagues^32^, further validating the potential for covalently conjugated trimer-liposomes for an HIV vaccine. BCR engagement at the cellular level is significantly enhanced through high multivalency of trimer antigens, leading to stronger germinal center responses and higher antigen-specific antibody titers (trimer^+^, V3^−^, 6xHis^−^) over immunization with soluble trimers in the context of ISCOMATRIX adjuvant. While the longevity of the antibody response was not reported by Bale *et al*. they did show that multiple immunizations continued to increase overall antigen-specific responses^32^. Further studies to understand how dosing and vaccination regimen can be manipulated to control humoral responses both in mice and in NHPs will be very beneficial to the HIV vaccine field. Finally, we fully explore the role of liposome composition in stability and quantitatively show the direct impact of increased serum stability on germinal center and antibody responses.

Though not evaluated here, liposomes also have the potential for the co-delivery of other antigens or adjuvants, to boost responses even further. These include, but are not limited to, additional T-cell help cues or cocktails of HIV env immunogens to increase both the overall strength and breadth of the immune response against HIV variants. Further studies to identify the optimal combination of trimers, particulate delivery, boosting regimen, and adjuvant choice will be required in pre-clinical models.

## Conclusion

Here we show direct evidence of improved MD39 SOSIP trimer-specific responses in mice by designing stabilized liposomes for oriented display of high-density trimers. When immunized with covalent MD39-coupled liposomes, mice had significantly higher GC and antigen-specific T_fh_ cell responses, resulting in significantly higher avidity serum MD39-specific IgG titers compared to those from immunization with soluble MD39. Particulate priming was found to be especially important in eliciting eventual high avidity antibody titers, regardless of boost immunization with either soluble or particulate trimers. These humoral responses were further improved by stabilizing liposomes with the incorporation of sphingomyelin. Together these results show promise for the use of HIV env-coupled liposomes as a potential basis of a prophylactic HIV vaccine.

## Materials and Methods

### Materials

Lipids 1,2-distearoyl-*sn*-glycero-3-phosphocholine (DSPC), 1,2-dipalmitoyl-*sn*-glycero-3-phosphocholine (DPPC), 1,2-dimyristoyl-*sn*-glycero-3-phosphocholine (DMPC), 1,2-dioleoyl-sn-glycero-3-[(N-(5-amino-1-carboxypentyl) iminodiacetic acid) succinyl] (nickel salt) (DGS-NTA(Ni)), 1,2-dioleoyl-sn-glycero-3-phosphoethanolamine-N-[4-(p-maleimidophenyl) butyramide] (sodium salt) (MPB) and porcine brain sphingomyelin were purchased from Avanti Polar Lipids (Alabaster, AL). Cholesterol was purchased from Sigma-Aldrich (St. Louis, MO). ISCOMATRIX was provided by CSL, Ltd. MPLA was purchased from Sigma-Aldrich (St. Louis, MO). Biotinylated 6xHis (Biotin-HHHHHH) and V3 (TRPNNNTRKSIRIGPGQAFYATG) linear peptides were purchased from Genscript (Piscataway, NJ).

### BG505 trimer and antibody production

BG505 SOSIP, MD39-6xHis, MD39-6xHis-Cys and MD39-StrepII were prepared as described previously^13,18^. Briefly, trimer genes were mixed with a plasmid containing furin protease (2 trimer: 1 furin ratio) and 293fectin, then transfected into FreeStyle 293F cells. After seven days, the trimers secreted from 293F cells were purified by affinity chromatography using HisTrap or StrepTrap HP columns (GE Healthcare) followed by size-exclusion chromatography (SEC) using a S200 Increase column (GE Healthcare) in PBS. The molecular weight of the trimer was confirmed by SEC multi-angle light-scattering using DAWN HELEOS II and Optilab T-rEX instruments (Wyatt Technology). Broadly neutralizing antibodies (VRC01, PGT121, PGT145, PGT151) and non-neutralizing antibodies (B6, 4025, 39F) were produced as IgG similar to the trimers, without the addition of furin protease. The antibodies were purified using a Capture Select IgG-CH1 column and dialyzed into PBS.

Murine VRC01 chimera antibody sequence was designed by substituting the constant heavy and light chains of human VRC01^47,48^ (obtained through the NIH AIDS Reagent Program, Division of AIDS, NIAID, NIH: CMVR VRC01 H, from Dr. John Mascola) with those from mouse IgG2c heavy chain and Ig kappa light chain. Chimeric sequences were synthesized as genomic blocks (Integrated DNA Technologies) and cloned into gWIZ expression plasmids (Genlantis). Plasmids were transiently transfected into Expi293 cells (ThermoFisher Scientific) using a 2:3 ratio by mass of the heavy chain and light chain plasmids. Cell culture supernatants were collected at 5 days post-transfection and antibody was purified in a ÄKTA pure chromatography system using HiTrap Protein A affinity columns (GE Healthcare Life Sciences).

### Covalent trimer-conjugated liposome synthesis and characterization

MD39-conjugated liposomes were prepared as previously described with some modifications^20^. Briefly, unilamellar liposomes comprised of phospholipid:cholesterol:DGS-NTA(Ni):MPB lipids in a 61.5:28.5:5:5 mole ratio were synthesized by lipid film rehydration and membrane extrusion using a 100 nm membrane at T > T_m(phospholipid)_ (DMPC = 37 °C, DPPC = 50 °C, DSPC = 60 °C), followed by post-synthesis binding of 6xHis-Cys C-terminal-modified trimer (MD39-HHHHHHC) for 1 hour at 37 °C in PBS (final concentrations 2.1 µM MD39, 3.53 mM liposomes) followed by 16–18 hr incubation at 4 °C with rotation. Non-covalent liposomes were prepared similarly without MPB lipid (phospholipid:cholesterol:DGS-NTA(Ni) lipids in a 66.5:28.5:5 mole ratio). Increasingly stabilized liposomes were prepared with 15 mole% MPB lipid and with sphingomyelin in place of cholesterol (phospholipid:sphingomyelin:DGS-NTA(Ni):MPB lipids in a 51.5:28.5:5:15 mole ratio). For all preparations, unconjugated trimer was removed by size exclusion chromatography after coupling using Sepharose CL-2B resin (Sigma).

Total conjugated trimer was quantified by ELISA in the presence of 1% triton-X and 100 mM imidazole to fully disrupt liposomes and Ni-6xHis interactions, respectively. Trimer was captured on Nunc MaxiSorp plates coated with 2 µg/ml (mouse Fc) VRC01 and detected using 0.2 µg/ml PGT151, followed by secondary detection with 1:5000 goat anti-human IgG-HRP conjugate. Antigenic profiles were similarly determined by ELISA on intact liposomes captured on VRC01-coated plates. Trimer density was calculated using a phospholipid quantification assay (Sigma) to determine the theoretical number of monodisperse, unilamellar liposomes as previously described^18^. Trimer-conjugated liposomes were also characterized by dynamic light scattering and cryoelectron microscopy (Jeol 2100 F TEM) in the Swanson Biotechnology Center Nanotechnology Core at the Koch Institute, MIT.

### Liposome serum stability analysis

Trimer-conjugated liposomes were incubated with 10–20% (final concentration) normal mouse serum or PBS (control) for 1–3 days at 37 °C after which released trimer was separated from liposomes by size exclusion chromatography using Sepharose CL-2B resin. Intact trimer in each elution fraction was quantified using PGT151 as the detection antibody as described above. For large liposome comparison studies, all samples were initially only run once (n = 1). For all other direct comparison studies, all samples were run in triplicate (n = 3).

### *In vitro* B cell activation

VRC01 BCR-expressing Ramos Burkitt’s lymphoma B cells were a gift from Daniel Lingwood at the Ragon Institute of MGH, MIT, and Harvard. Cells were stained with 10 µM of the calcium indicator fluo-4 (Thermo Fisher) for 30 minutes in serum-free RPMI prior to activation. Next, 250 µl cells at 1 × 10^6^ cells/ml were stimulated with either MD39 or MD39-liposomes at indicated concentrations and fluo-4 signal was measured by flow cytometry. Cells were pre-warmed to 37 °C and then read for 45 seconds prior to addition of MD39 to set a baseline and then read for 4 additional minutes. All flow cytometry was carried out on a BD LSR II in the Swanson Biotechnology Center Flow Cytometry Core at the Koch Institute, MIT. For samples where activation was measured for longer periods (>5 minutes), fluorescence change over time was measured directly using a Tecan fluorescent plate reader. Fluorescent signal was normalized to the baseline signal for each sample. All experiments were repeated at least twice.

### Synthesis of saponin adjuvant

For some studies, an in-house formulation of a saponin adjuvant was utilized. The saponin adjuvant closely resembled ISCOM-like nanoparticles comprised of self-assembled cholesterol, phospholipid, and Quillaja saponin and was prepared as previously described^49^; all synthesis was performed under sterile conditions with sterile reagents. Briefly, 10 mg each of cholesterol (Avanti Polar Lipids 700000) and DPPC (Avanti Polar Lipids 850355) were dissolved separately in 20% MEGA-10 (Sigma D6277) detergent at a final concentration of 20 mg/ml and 50 mg Quil-A saponin (InvivoGen vac-quil) was dissolved in MQ H_2_O at a final concentration of 100 mg/ml. Next, DPPC solution was added to cholesterol followed by addition of Quil-A saponin in rapid succession and the volume was brought up with PBS for a final concentration of 1 mg/ml cholesterol and 2% MEGA-10. The solution was allowed to equilibrate at 25 °C overnight, followed by 5 days of dialysis against PBS using a 10k MWCO membrane. The adjuvant solution was then filter sterilized using a 0.2 µm Supor syringe filter, concentrated using 50k MWCO centricon filters, and further purified by FPLC using a Sephacryl S-500 HR size exclusion column. Each adjuvant batch was finally characterized by negative stain TEM and DLS to confirm uniform morphology and size and validated for low endotoxin by Limulus Amebocyte Lystae assay (Lonza QCL-1000). Final adjuvant concentration was determined by cholesterol quantification (Sigma MAK043).

### Immunizations in mice

All procedures used in animal studies were approved by the Committee on Animal Care at the Massachusetts Institute of Technology following local, state, and federal regulations. Female balb/c mice (6–10 weeks old, Jackson Laboratories) were immunized with 1 µg MD39 trimer either in soluble form or conjugated to liposomes mixed with 0.2 U ISCOMATRIX adjuvant (CSL Ltd.) or 5 µg in-house saponin adjuvant, unless otherwise noted. For preliminary analysis of V3-specific responses, mice were similarly immunized with 1 µg wild-type BG505 SOSIP either in soluble form or conjugated to liposomes mixed with 1 µg MPLA (either in soluble form or directly in SOSIP liposomes). Liposomes containing MPLA were prepared exactly as above by adding MPLA along with the other lipids in the organic solution prior to drying the lipid film (final 0.4 µg/µl MPLA in starting 6.45 mM liposome preparation). All mice were immunized subcutaneously with 50 µl trimer/adjuvant solution in PBS on each side of the tail base. For long-term MD39 trimer immunizations, mice were primed on day 0 and boosted at 6 weeks, with weekly retro-orbital blood collection. Blood was collected into serum separator tubes and centrifuged at 10,000 × g for 5 min at 4 °C. Sera was stored at −80 °C until analysis.

### Germinal center and T_fh_ analysis

Mice were sacrificed by CO_2_ inhalation and both inguinal lymph nodes were harvested at specified days. Lymph nodes were processed into single-cell suspensions using enzymatic digestion with 0.8 mg/ml Collagenase/Dispase and 0.1 mg/ml DNAse (Roche Diagnostics) in complete RPMI (with 10% FBS and antibiotics) at 37 °C, followed by passage through a 70-µm cell strainer (BD Biosciences). For germinal center analysis, cells were washed with PBS and stained with Live/Dead Aqua (Life Technologies) for 15 minutes at 25 °C. Samples were then treated with anti-CD16/32 Fc block (BioLegend), followed by staining with anti-CD3e-PerCP-Cy5.5 (BD Biosciences), anti-B220-PE-Cy7 (eBioscience), anti-CD138-PE (BD Biosciences), anti-IgD-APC (eBioscience), anti-GL7-FITC (BD Biosciences), and PNA-biotin (VectorLabs) + streptavidin-APC-Cy7 (eBioscience) in PBS/1% BSA and finally fixed and stored at 4 °C until analysis. In parallel, antigen-specific T_fh_ analysis was performed as previously described^39^. Briefly, 1 × 10^6^ cells were stimulated with 5 µg/ml of a BG505 SOSIP-derived overlapping peptide pool for 18 hours at 37 °C. Unstimulated cells were run as a control. Cells were then washed and similarly stained with Live/Dead Aqua and treated with anti-CD16/32 Fc block, followed by staining with anti-B220-BV510 (BD Biosciences), anti-CD4-PerCP-Cy5.5 (eBioscience), anti-CD44-AlexaFluor700 (BioLegend), anti-PD1-PE-Cy7 (eBioscience), anti-OX40-APC (BioLegend), anti-CD25-FITC (BioLegend), anti-PDL1-BV421 (BioLegend), and purified rat anti-CXCR5 (BD Biosciences) + goat anti-rat IgG-biotin (Jackson Immunoresearch) + streptavidin-PE (BioLegend) in PBS/2% normal mouse serum/2% FBS/1% BSA and finally fixed and stored at 4 °C until analysis. Flow cytometry was carried out on a BD LSR Fortessa.

### Serum ELISAs

Serum anti-BG505 MD39 titers were quantified by ELISA using Nunc MaxiSorp plates through a sandwich ELISA designed to preserve MD39 tertiary structure. Plates were coated with 1 µg/ml rabbit anti-6xhis (Genscript), blocked with milk block (PBS/5% skim milk/10% goat serum/1% FBS/1% BSA/0.2% Tween-20) overnight at 4 °C, and then incubated with 2 µg/ml MD39-6xhis for 2 hours. Mouse sera was diluted in milk block starting at 1:50 with 4x serial dilutions and incubated for 2 hours, followed by detection with 1:5000 goat anti-mouse IgG-HRP conjugate in milk block. For MD39 titer analysis that excluded histag responses, plates were instead coated with 2 µg/ml rabbit anti-strepII (Novus Biologicals), blocked with PBS/1% BSA overnight at 4 °C, and then incubated with 2 µg/ml MD39-strepII. Mouse sera was diluted in PBS/1% BSA starting at 1:50 with 4x serial dilutions and incubated for 2 hours, followed by detection with 1:5000 goat anti-mouse IgG-HRP conjugate for 1 hour. To determine avidity, ELISAs were conducted similarly using rabbit anti-6xhis to capture MD39-his, since the anti-strepII/MD39-strepII interaction was itself disrupted in the presence of urea and could not be used for this analysis. Following serum incubation, plates were treated with 4–5 M urea in PBS or PBS alone for 10 minutes at 25 °C followed by several washes before detection. Urea and PBS treatment for each individual sample was always conducted on the same plate. Avidity indices were calculated as the 100*[(titers in the presence of urea)/(titer in the absence of urea)]. Additionally, histag titers were determined by capturing a biotinylated 6xHis peptide at 2 µg/ml onto streptavidin coated plates while V3 titers were determined by directly coating V3 linear peptide at 2 µg/ml. For both histag and V3 ELISAs, PBS with 1% BSA was used to block and as the diluent for serum and detection antibodies. For all titer analyses, all samples being directly compared were run at the same time. All titers reported are inverse dilutions where A_450nm_ − A_540nm_ (reference wavelength) equals 0.3.

### Statistics

Statistical analyses were performed using GraphPad Prism software. All values and error bars are shown as mean ± standard deviation, with the exception of serum titer data that are shown as mean ± 95% confidence interval. Titers were analyzed using either the Mann-Whitney test for direct comparisons or a Kruskal-Wallis test followed by a Dunn’s post-test for multiple groups. All other data were analyzed using a two-tailed unpaired *t* test to determine statistical significance between two experimental groups or a one-way ANOVA followed by a Tukey post-test to compare multiple groups, unless otherwise noted.

## Electronic supplementary material

Supplementary Text and Figures

## Data Availability

All data generated and analyzed during this study are included in this published article (and its Supplementary Information files).
