# Supplementary material for: Enhancing Humoral Responses Against HIV Envelope Trimers via Nanoparticle Delivery with Stabilized Synthetic Liposomes
Source: Sci Rep. 2018 Nov 8;8:16527. doi: 10.1038/s41598-018-34853-2 (PMC6224390; doi:10.1038/s41598-018-34853-2)
Supplement: Supplementary file 1 — Supplementary Text and Figures [file 41598_2018_34853_MOESM1_ESM.docx]

**Supplemental Information**

**Enhancing Humoral Responses Against HIV Envelope Trimers via Nanoparticle Delivery with Stabilized Synthetic Liposomes**

***Talar Tokatlian^1^, Daniel W. Kulp^2,3,4^, Andrew A. Mutafyan^1^, Christopher A. Jones^1^, Sergey Menis^2,3^, Erik Georgeson^2,3^, Mike Kubitz^2,3^, Michael H. Zhang^1^, Mariane B. Melo^1^, Murillo Silva^1^, Dong Soo Yun^1^, William R. Schief^2,3,5,7^, Darrell J. Irvine^1,3,6,7,8,*^***

^1^Koch Institute, MIT; ^2^International AIDS Vaccine Initiative, The Scripps Research Institute; ^3^Center for HIV/AIDS Vaccine Immunology and Immunogen Discovery, The Scripps Research Institute; ^4^Vaccine and Immunotherapy Center, The Wistar Institute; ^5^Immunology and Microbial Science, TSRI; ^6^Dept. of Biological Engineering and Materials Science & Engineering, MIT; ^7^Ragon Institute of MGH, MIT, and Harvard; ^8^Howard Hughes Medical Institute

*Corresponding author (djirvine@mit.edu)

Contains supplemental figures 1-8.

**Supplemental Figure 1:** Representative size exclusion chromatography profile of trimer-conjugated liposomes after incubation in PBS (control) or serum. Trimer on liposomes (~150nm) and released/free trimers elute at distinct times using Sepharose CL-2B resin.


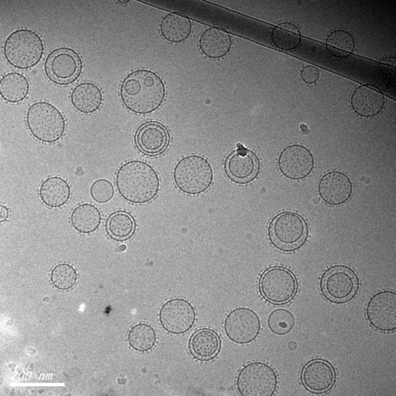

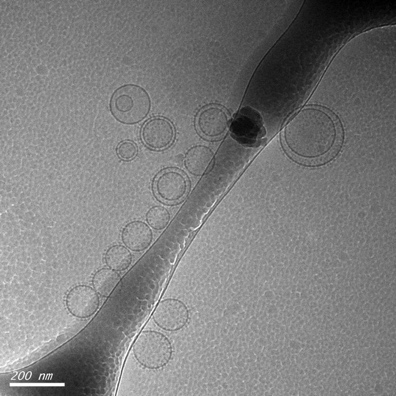

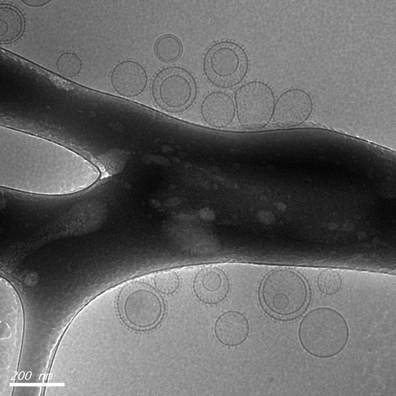

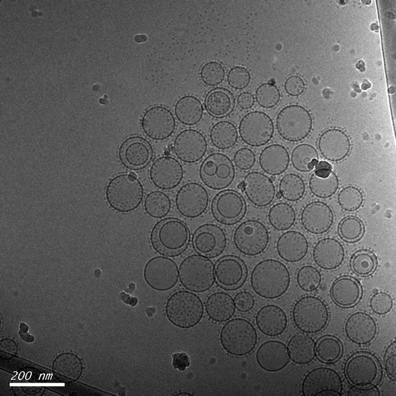

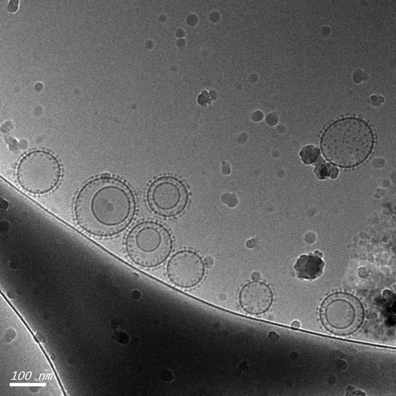

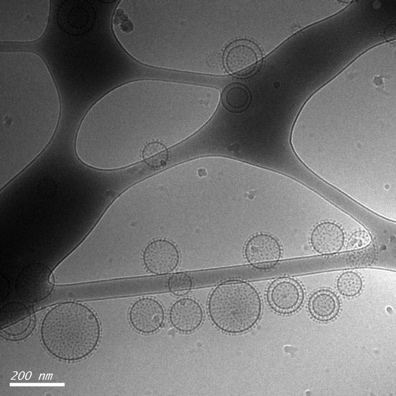


Ni only lipos (various lipid compositions)

Ni/MPB lipos (various lipid compositions)

High density (1.0x)

Med. density (0.5x)

Low density (0.2x)

**A**

**C**

**B**

**D**


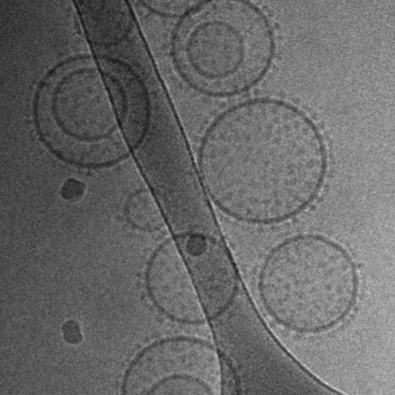


***100nm***


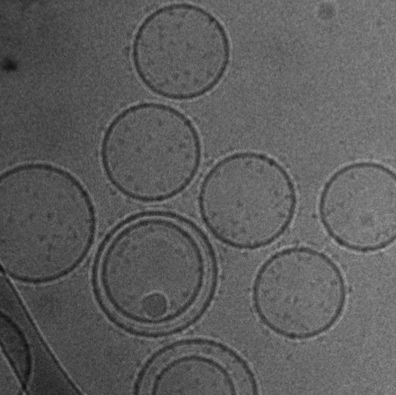


***100nm***


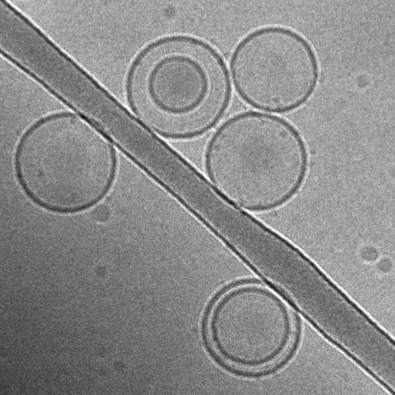


***100nm***

**Supplemental Figure 2:** Additional characterization of trimer-conjugated liposomes. Representative cyroEM of (A) non-covalent and (B) covalent MD39 conjugated liposomes from 3 separate preparations. (C) Representative cryoEM of covalent MD39-trimer conjugated liposomes at various surface densities. Relative trimer concentration during liposome conjugation denoted within parentheses. (D) DLS size distribution of covalent MD39 conjugated liposomes.

**Supplemental Figure 3:** Humoral responses in mice over time comparing immunization with MD39 trimers either in soluble form (red) or on covalent liposomes (blue). Mice were immunized with 1 µg MD39 trimer and ISCOMATRIX, and boosted at 6 weeks. Serum MD39-specific titers determined by ELISA using non-6xhis tagged MD39 trimers to exclude histag-specific responses (n=5 animals/group).

**A**

**B**

**Supplemental Figure 4:** Humoral responses in mice 3 weeks post-boost comparing immunization with MD39 trimers either in soluble form or on non-covalent or covalent liposomes. Mice were immunized with 1 µg MD39 trimer and ISCOMATRIX, and boosted at 6 weeks. (A) Serum MD39-6xhis-specific titers (n=8-12 animals/group). (B) Serum 6xhis tag-specific titers. Dotted lines denote limit of detection. Titers were analyzed by a Kruskal-Wallis test.

**A**

**B**

**Supplemental Figure 5:** (A) Serum V3-specific titers following immunization with 1 µg MD39 trimers and ISCOMATRIX (n=8-12 animals/group); dotted line denotes limit of detection. (B) Negative (VRC01) and positive (14e, 39F) V3-specific monoclonal antibody binding to V3 peptide are shown for reference.

**A**

**B**

**Supplemental Figure 6:** Humoral responses in mice 3 weeks post-boost comparing immunization with MD39 trimers either in soluble form or on fluid DMPC or rigid DSPC covalent liposomes. Mice were immunized with 1 µg MD39 trimer and ISCOMATRIX, and boosted at 6 weeks. (A) Serum MD39-6xhis-specific titers (n=8-12 animals/group); dotted line denotes limit of detection. (B) Avidity of polyclonal antibodies. Avidity was analyzed using independent unpaired *t* tests for direct comparisons.

**A**

**B**

**Supplemental Figure 7.** GC responses 7 days following immunization with 5 µg in-house formulated saponin adjuvant and blank DSPC liposomes or DSPC liposomes bearing 5% Ni-NTA functionalized lipids. Frequencies of (A) GL7+ PNA+ IgD-low (B220+) GC B cells and (B) total (CXCR5+ PD1+ with parent gate CD4+ CD44+) in draining inguinal lymph nodes (*n*=4-5 animals/group). Relative responses were analyzed by an ordinary one-way ANOVA with Tukey post-test.

**A**

**B**

**Supplemental Figure 8:** Stability characterization of various covalent MD39 trimer-conjugated liposome formulations. Serum stability analysis comparing liposomes with (A) increasing mole % MPB or MCC lipids or with the addition of PEG lipids or (B) increasing mole % MPB, incorporation of saturated MPB (MPB(16:0)), and exchange of cholesterol for sphingomyelin each with DSPC, DPPC, or DMPC as the base structural lipid all relative to the original formulation (red; 61.5:28.5:5:5 DSPC:cholesterol:Ni-NTA:MPB) after 3 days in 20% serum at 37^o^C. For all formulations tested, 5 mole % Ni-NTA and 28.5 mole % cholesterol or sphingomyelin was maintained even as concentration of maleimide lipid varied. Structural phospholipid content was adjusted to maintain equivalent total moles of lipid between formulations. For such preliminary comparison studies, all samples were initially only run once (n=1).
